# Longitudinal relationship between anxiety symptoms and occupational burnout dimensions among newly hired nurses: a cross-lagged network analysis

**DOI:** 10.3389/fpsyt.2026.1884139

**Published:** 2026-08-07

**Authors:** Zhilin Bian, Linglin Guo, Mingxia Li, Zi Yang, Yanhong Cai, Feng Wang, Xiaozhen Song, Qian You, Juan Chen, Peiyuan Qiu

**Affiliations:** 1 Department of Epidemiology and Statistics, West China School of Public Health and West China Fourth Hospital, Sichuan University, Chengdu, China; 2 Mental Health Center, West China Hospital of Sichuan University, Chengdu, China; 3 West China School of Nursing, Sichuan University, Chengdu, China; 4 Thoracic Oncology Ward, Cancer Center, West China Hospital of Sichuan University, Chengdu, China

**Keywords:** anxiety symptoms, cross-lagged network analysis, longitudinal study, nurses, occupational burnout

## Abstract

**Introduction:**

This study aimed to examine the symptom networks of anxiety and occupational burnout among newly hired nurses and to identify central, bridge, and longitudinally influential anxiety symptoms or burnout dimensions that may inform targeted interventions.

**Methods:**

Data were collected from newly hired nurses at a tertiary hospital in China between July 2023 and July 2024. Anxiety and burnout were assessed using the Generalized Anxiety Disorder-7 and Maslach Burnout Inventory -Human Services Survey, respectively. Contemporaneous networks at three time points and cross - lagged panel models (for longitudinal networks) were built, with centrality indices evaluating network features.

**Results:**

1) Anxiety prevalence: 4.3%–14.4%; burnout detection rate: 47.4%–54.7%. 2) Contemporaneous networks: “trouble relaxing”, “uncontrollable worry”, “afraid something will happen” served as the central anxiety symptoms at the three time points, respectively; “depersonalization”, “emotional exhaustion” were central burnout dimensions, with “emotional exhaustion” having highest bridge strength. 3) Occupational burnout dimensions showed high autoregressive coefficients. 4) Temporal networks showed that from T1 to T2, temporal associations were mainly concentrated within anxiety symptoms, with “uncontrollable worry” and “worry too much” showing the highest in-strength value. From T2 to T3, stronger associations emerged between anxiety and burnout, with the strongest edge from emotional exhaustion to later nervousness or anxiety; within burnout dimensions, lower personal accomplishment was associated with higher subsequent depersonalization, and depersonalization was related to later emotional exhaustion.

**Conclusion:**

Worry-related symptoms, irritability, emotional exhaustion, and depersonalization among newly hired nurses played important roles in the networks. Early screening and targeted interventions should focus on reducing anxiety and uncertainty in the early stage, while greater attention should be paid to occupational burnout as newly hired nurses gradually adapt to clinical work.

## 1 Introduction

Nurses, as indispensable members of healthcare teams, directly engage in patient-centered care, substantially impacting recovery-related clinical outcomes and shaping patient satisfaction levels (1). However, nurses face high risks of burnout due to prolonged stress, high work intensity and complex interpersonal relationships. The prevalence of burnout among nurses around the world is 11.23% (2), while it ranges from 50.0% to 64.5% in China (3). High burnout not only harms individual nurses’ health by triggering physical symptoms like fatigue and frequent illness, along with psychological distress and cognitive decline, but also threatens patient safety and healthcare system stability by reducing nursing quality, increasing medical errors, and raising turnover rates (2, 4). The WHO predicts a global nursing shortage of 7.6 million by 2030, with burnout driving attrition (5). A 2008 U.S. survey showed 35% of non-practicing employed nurses left due to burnout (2). High turnover strains healthcare budgets through recruitment and onboarding costs, undermining organizational stability.

Numerous studies have demonstrated a significant correlation between job burnout and anxiety (6, 7). And nurses exhibit particularly high anxiety levels compared to other healthcare workers (8). A 2020 meta-analysis showed a 25.8% anxiety prevalence among nurses, higher than 21.7% in doctors (9). Newly hired nurses, facing adaptation pressures in their early career, are especially vulnerable to anxiety, which may escalate into burnout if unmanaged. Liu F. et al. reported that anxiety not only directly impacts burnout, but also indirectly influences burnout through its association with depression in nurses (10). Additionally, Yuan, L. et al.’s study indicated that anxiety symptoms mediate the effects of work-related stress on job burnout among female nurses in China (11), while Turnipseed et al. found that burnout and anxiety symptoms are significantly interrelated, with the strongest link observed between anxiety and emotional exhaustion (7).

Although studies on anxiety and burnout among nurses have expanded in recent years, several methodological and theoretical gaps remain. Firstly, traditional latent variable models treat anxiety as a unidimensional construct, ignoring network-based associations between burnout dimensions and anxiety symptoms. Network theory of psychopathology proposes that mental health problems, such as anxiety, can be conceptualized as systems of dynamically interacting symptoms. Within such symptoms, specific symptoms may mutually reinforce one another and contribute to the maintenance of psychological distress (12). There have been some network analyses conducted separately on anxiety and burnout. Network analyses of anxiety in Chinese healthcare workers have identified “trouble relaxing” and “uncontrollable worry” as central symptoms, differing from “irritable” in adolescents or “worry” in nursing students (13–16). For burnout, a Chinese nurse study found strong associations between emotional exhaustion and depersonalization, with “feeling emotionally drained” and “becoming callous toward work” as key nodes (17). A cross-lagged study of primary school teachers showed that emotional exhaustion and personal accomplishment at time 1 predicted the same at time 2, while depersonalization at time 1 predicted depersonalization and personal accomplishment at time 2 (18).

Secondly, most existing studies have examined the relationship between anxiety and burnout using cross-sectional designs. However, these psychological problems may influence each other over time, particularly during the critical period of career adaptation for new nurses. To fill this gap, the present study employs cross-lagged panel network (CLPN) models on longitudinal data. CLPN models serve to construct temporal network relationships between individual symptoms, and they are vital for examining temporal and directional links across variables within longitudinal data (19). This approach provides powerful and robust inferences at the symptom level, advancing our understanding of the longitudinal dynamic interaction mechanisms between anxiety symptom clusters and the three dimensions of burnout.

Therefore, this study aimed to: 1) identify central and bridge items of anxiety symptoms and burnout dimensions among newly hired nurses using cross-sectional network analysis; 2) examine temporal symptom associations using cross-lagged panel network analysis. Findings aim to inform interventions to reduce anxiety and burnout in this population.

## 2 Methods

### 2.1 Study design and participants

This study sampled newly hired nurses from a tertiary general hospital in Sichuan Province, China. Data collection occurred at three time points with 6-month intervals: Time 1 (T1) in July 2023 (upon the nurses’ hospital enrollment), Time 2 (T2) in January 2024, and Time 3 (T3) in July 2024. After providing informed consent, participants completed the questionnaires online via Wenjuanxing, a widely used web-based survey platform in China. All collected questionnaires were uniformly coded, with dedicated personnel responsible for sorting and storage. The inclusion criteria for this study were: (a) holding a valid nursing license and practitioner certificate; (b) newly hired to the hospital; and (c) willingness to participate in follow-up assessments.

**Figure 1:**
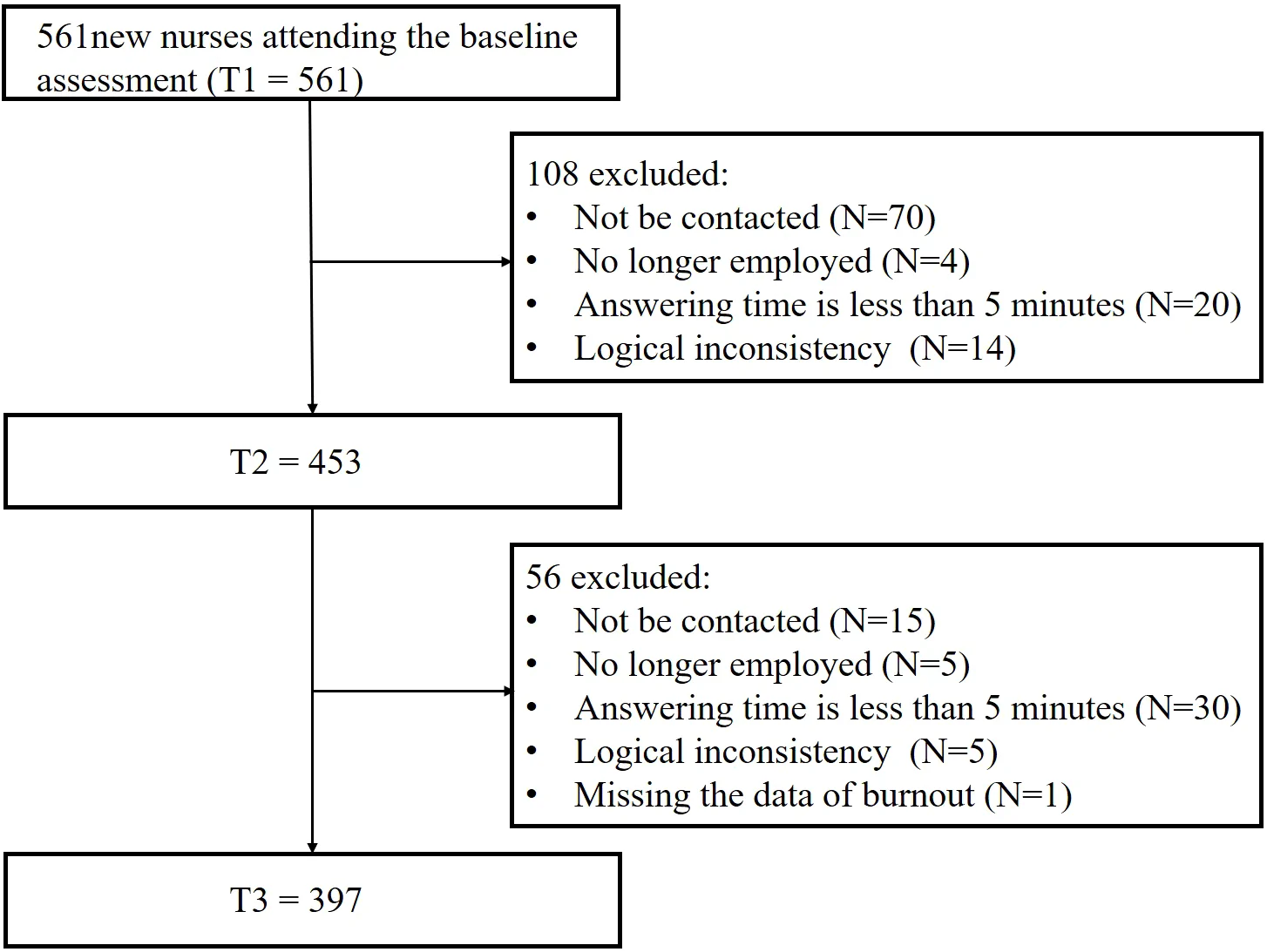
Flowchart for the selection of the analyzed study sample.

A total of 561 newly hired nurses participated in the baseline survey, and they were excluded if they met any of the following criteria: (a) questionnaires were invalid for response time < 5 minutes, identical responses to >90% of items, or logical inconsistencies in answers; (b) working in non-clinical positions (e.g., sterile supply rooms); (c) having a diagnosed mental or neurological disorder; or (d) missing data on anxiety or burnout in both the baseline survey and two follow-up surveys. After exclusion in accordance with the above criteria, a total of 397 nurses were finally included in the study (**Figure 1**), including 62 males (15.6%), 335 females (84.4%), with an average age of 22.43 ± 0.96 years. This study was reported with reference to the STROBE reporting guideline.

### 2.2 Measures

#### 2.2.1 Anxiety symptoms

Anxiety was assessed using the Generalized Anxiety Disorder-7 (GAD-7), a 7-item scale evaluating symptoms over the past two weeks (20). Items include “nervousness or anxiety”, “uncontrollable worry”, “worry too much”, “trouble relaxing”, “restlessness”, “irritable”, “afraid something will happen”, rated on a 0–3 Likert scale with total score ranging from 0 to 21. Scores were categorized as minimal anxiety (0–4), mild anxiety (5–9), moderate anxiety (10–14), and severe anxiety (15–21). In this study, the *Cronbach’s α* coefficients for GAD-7 at T1, T2 and T3 were 0.796, 0.879, and 0.844 respectively.

#### 2.2.2 Occupational burnout

Burnout was assessed by the Maslach Burnout Inventory - Human Services Survey (MBI-HSS) (21), which was translated into Chinese and validated by Feng Ying et al. (22). The scale consists of three dimensions with a total of 22 items, including emotional exhaustion (9 items), depersonalization(5 items), and personal accomplishment (8 items). A 7-point Likert scale was used (0 = never, 6 = every day). Emotional exhaustion and depersonalization were scored such that higher scores indicated more severe burnout. The personal accomplishment dimension was analyzed using its original scoring direction, with lower scores indicating lower personal accomplishment and more severe burnout. For descriptive classification, each participant was first coded as having an abnormal score on each of the three MBI-HSS dimensions according to the established cutoffs: emotional exhaustion ≥27, depersonalization ≥8, and personal accomplishment ≤24 (23). Burnout severity was then classified according to the number of abnormal dimensions: none = 0 abnormal dimensions, mild = 1 abnormal dimension, moderate = 2 abnormal dimensions, and severe = 3 abnormal dimensions. These four categories were mutually exclusive, and each participant was assigned to only one category at each time point. The *Cronbach’s α* coefficients of the scale in this study were 0.855, 0.891, and 0.896 at T1, T2, and T3, respectively.

#### 2.2.3 Covariates

Baseline demographic variables including gender, age, education level, and marital status were incorporated as covariates in the temporal network analyses. Age was treated as a continuous covariate, whereas sex, education level, and marital status were dummy-coded before entering the models. These covariates were included as adjustment variables in the nodewise regularized regressions but were not displayed as nodes in the final temporal networks.

### 2.3 Statistical analysis

This study used data on anxiety symptoms and burnout collected at baseline (T1) and two follow-up time points (T2 and T3). For descriptive analyses, anxiety severity was classified according to the total GAD-7 score, whereas burnout severity was classified using the cutoffs for the three MBI-HSS dimensions. For network analyses, the seven individual GAD-7 items and the three MBI-HSS dimensions were used as 10 nodes. Cross-sectional network analyses were performed separately at each time point, followed by longitudinal analyses examining the temporal associations from T1 to T2 and from T2 to T3. Participants with complete anxiety and burnout data across the three waves were included in the main analyses. No item-level missing values in anxiety symptoms or burnout dimensions were imputed, and no wave-level missing data were imputed. Mean imputation was applied only to two missing age values, as age was a covariate with a narrow range. All analyses were conducted in RStudio 4.3.2.

#### 2.3.1 Cross-sectional network analysis

Contemporaneous networks were built via Gaussian Graphical Models (GGMs) with partial correlation analysis, using the bootnet and qgraph packages (24). The Extended Bayesian Information Criterion and graphical Least Absolute Shrinkage and Selection Operator (EBICgLASSO) were used for optimal sparse estimation of partial correlation matrices (25). In network structure graphs, nodes represented symptoms, edges represented partial correlations, and thicker edges indicated stronger associations (26). In the connections between nodes, blue edges are set to represent positive correlations, while red edges indicate negative correlations.

To evaluate the structural importance of each node in the network, the centralityPlot function in the qgraph package was used to estimate and visualize three centrality indices of symptoms (27). For a certain node, the more edges connected to it and the greater the weight values of the edges, the stronger the centrality of the node. Central symptoms were identified primarily based on node strength. Bridge symptoms, potentially serving as “bridges” between different symptom clusters to facilitate the interaction or co-occurrence of symptoms, mainly used the Bridge Strength index as the judgment basis.

The robustness of the network model was evaluated by calculating the correlation stability (CS) coefficients of centrality indices using case-dropping bootstrap methods (28), which were implemented via the bootnet package in R. CS values exceeding 0.70 denote excellent stability, those between 0.50 and 0.70 indicate acceptable stability, and the minimum threshold is set at 0.25. Additionally, network comparison tests (NCT) were performed using the NetworkComparisonTest package in R to analyze contemporaneous network structures and determine whether significant differences existed between distinct networks (29).

#### 2.3.2 Cross-lagged panel network analysis

Cross-lagged panel network analysis constructed cross-lagged network models for two time periods: T1 (baseline) to T2 (first follow-up) and T2 to T3 (second follow-up). In the cross-lagged network structure, nodes represent individual GAD-7 items or the three MBI-HSS dimensions, the direction of arrows on connections indicates the effect direction between nodes, with blue arrows denoting positive predictive effects and red arrows indicating negative predictive effects (30).

For each temporal network, each node at the later time point was regressed on all 10 nodes at the previous time point. Autoregressive paths (paths from a node to itself across adjacent waves) were included and penalized like other cross-lagged paths. For clarity, they were not shown in the main figures and were presented in **Supplementary Figure 1**. Sex, age, education level, and marital status were included as covariates in each nodewise regression. Age was treated as a continuous variable and standardized, whereas sex, education level, and marital status were dummy-coded. These covariates were included for statistical adjustment but were not displayed as nodes in the final networks.

To address scale differences between GAD-7 items and MBI-HSS dimensions, all node variables were z-standardized before model estimation. The models were estimated using least absolute shrinkage and selection operator regularized linear regression with the glmnet package in R. For each nodewise regression, the penalty parameter was selected using 10-fold cross-validation. The main analysis used lambda.1se to obtain a more parsimonious network, and sensitivity analyses were conducted using lambda.min and models without demographic covariates. Because family = “gaussian” was specified in glmnet, GAD-7 items and MBI-HSS dimensions were treated as continuous variables in the temporal network models.

Meanwhile, the study used the centralityPlot function in R packages to calculate the in-prediction and out-prediction centrality indices for each node (30). In-prediction reflected a node’s susceptibility to prior symptoms, while out-prediction reflected its predictive power for subsequent symptoms. Network accuracy and stability were assessed via case-dropping bootstrapping, with CS > 0.5 indicating acceptable stability. Sensitivity analyses were conducted to assess the effects of covariate adjustment and penalty selection.

## 3 Results

### 3.1 Descriptive statistics

**Figure 2:**
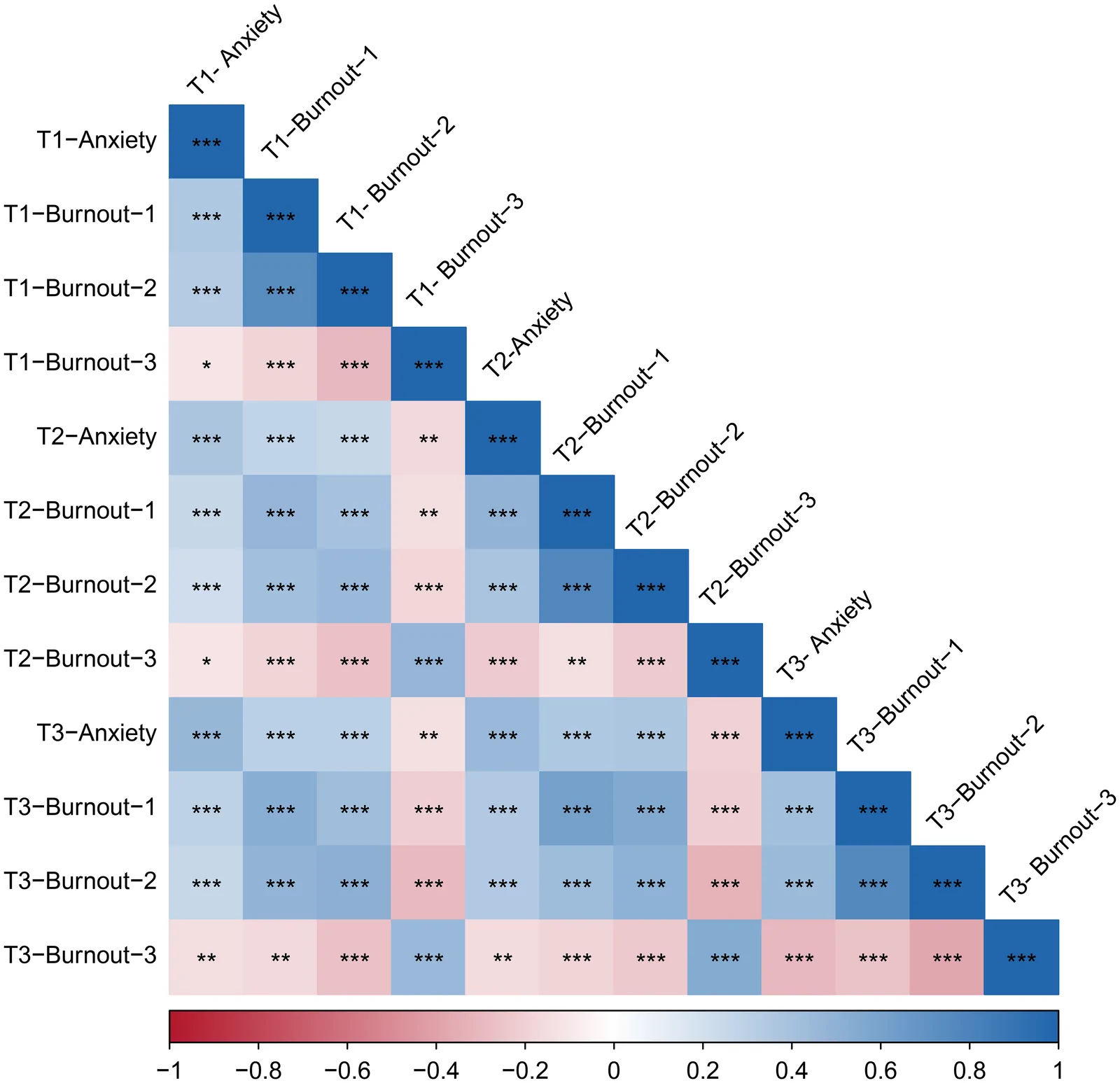
Correlation heatmap of study variables† (N = 397). *** *p* < 0.001; ** *p* < 0.01; * *p* < 0.05. Burnout-1= emotional exhaustion; Burnout -2= depersonalization; Burnout -3= personal accomplishment (Personal accomplishment was analyzed using its original scoring direction; therefore, lower scores indicate lower personal accomplishment and greater burnout.).

Among the 397 newly hired nurses, the majority (99.5%) held a bachelor’s degree, and 73.0% were single (**Table 1**). The prevalence of anxiety was 4.3% at T1, 14.4% at T2, and 11.1% at T3. The proportions of mild burnout were 37.0% (n =147), 33.5% (n = 133), and 29.2% (n = 116) at T1, T2, and T3, respectively. The corresponding proportions of moderate-to-severe burnout were 14.3% (n=57), 21.2% (n=84), and 18.1% (n = 72) (**Table 2**). The continuous descriptive statistics for the GAD-7 total score and the three MBI-HSS dimensions at T1, T2, and T3 were listed in **Supplementary Table 1**. **Figure 2** presents the correlation heatmap of all study variables. Anxiety showed significant positive correlations with emotional exhaustion and depersonalization, as well as a significant negative correlation with personal accomplishment; exact correlation coefficients were listed in **Supplementary Table 2**.

**Table 1:** Baseline characteristics of the sample.

Characteristics	Frequency (%)/ $\bar{x}$ (SD)
Age	22.43 (0.96)
Gender	
Male	62 (15.6%)
Female	335 (84.4%)
Education	
Junior college education or below	2 (0.5%)
Bachelor’s degree	395 (99.5%)
Marriage	
Single	290 (73.0%)
Unmarried and in a romantic relationship	106 (26.7%)
Married	1 (0.3%)

**Table 2:** Levels of anxiety and burnout among study participants.

Variable/time point	None, n (%)	Mild, n (%)	Moderate, n (%)	Severe, n (%)
Anxiety				
T1	380 (95.7%)	17 (4.3%)	0 (0.0%)	0 (0.0%)
T2	340 (85.6%)	55 (13.9%)	2 (0.5%)	0 (0.0%)
T3	353 (88.9%)	42 (10.6%)	2 (0.5%)	0 (0.0%)
Burnout				
T1	193 (48.6%)	147 (37.0%)	51 (12.8%)	6 (1.5%)
T2	180 (45.3%)	133 (33.5%)	75 (18.9%)	9 (2.3%)
T3	209 (52.6%)	116 (29.2%)	64 (16.1%)	8 (2.0%)

### 3.2 Cross-sectional networks of anxiety and burnout at three time points

**Figure 3:**
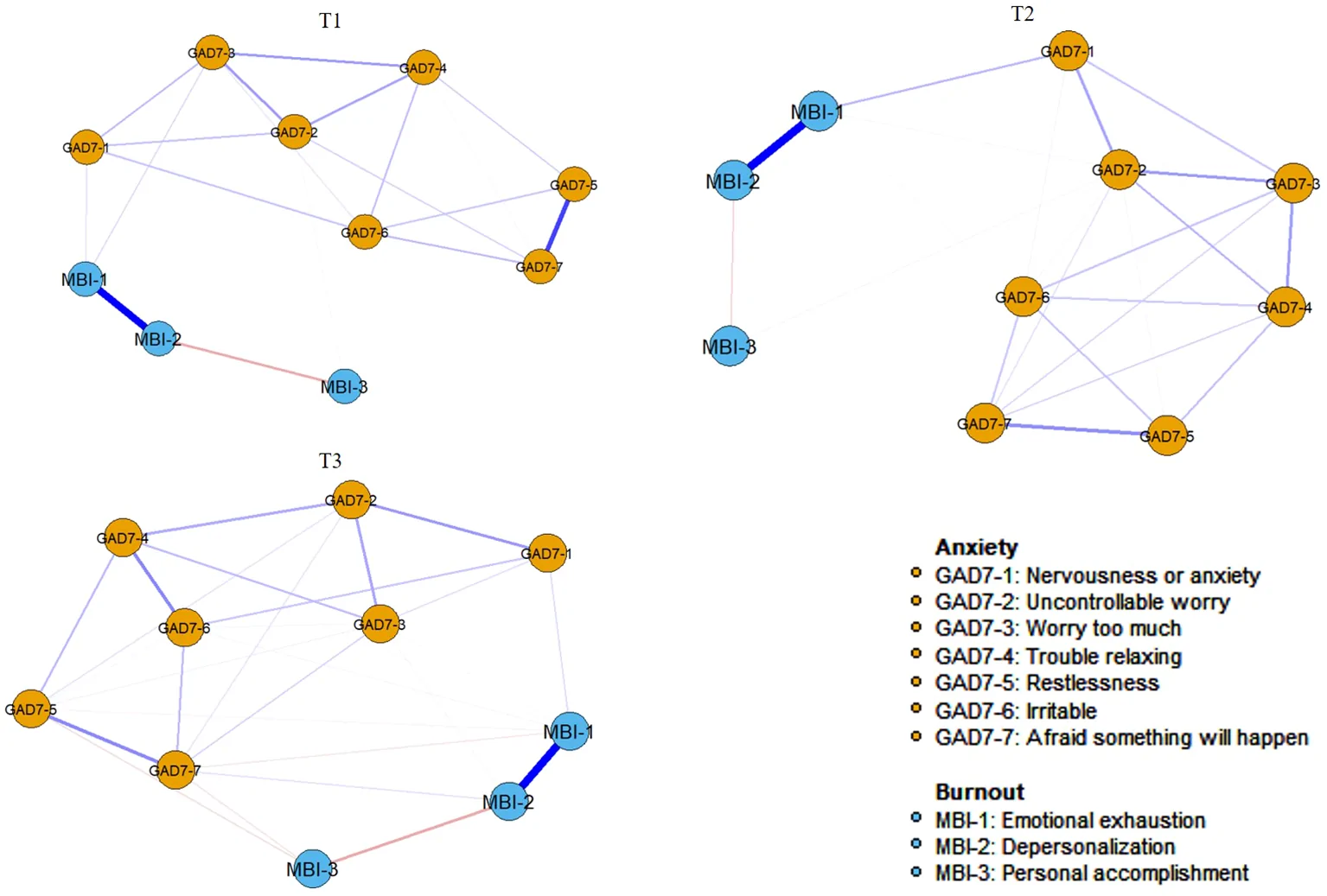
Contemporaneous networks of anxiety symptoms and burnout dimensions at T1 (July 2023), T2 (January 2024), and T3 (July 2024).

**Figure 3** depicts the contemporaneous networks of anxiety and burnout at T1, T2, and T3. For anxiety, “restlessness” and “afraid something will happen” showed strong associations at each time point (edge weights: *γ*_T1_ = 0.47, *γ*_T2_ = 0.30, *γ*_T3_ = 0.32). Similarly, “uncontrollable worry” was closely linked with “worry too much” (*γ*_T1_ = 0.25, *γ*_T2_ = 0.27, *γ*_T3_ = 0.24) and “trouble relaxing” (*γ*_T1_ = 0.23, *γ*_T2_ = 0.20, *γ*_T3_ = 0.22) across time. In addition, the association between “ uncontrollable worry “ and “nervousness or anxiety “ strengthened over time (*γ*_T1_ = 0.16, *γ*_T2_ = 0.24, *γ*_T3_ = 0.28), while the link between “ trouble relaxing “ and “ worry too much “ weakened (*γ*_T1_ = 0.26, *γ*_T2_ = 0.26, *γ*_T3_ = 0.18), and the association with “irritable” increased (*γ*_T1_ = 0.16, *γ*_T2_ = 0.15, *γ*_T3_ = 0.32). For burnout, emotional exhaustion and depersonalization exhibited the strongest connections (*γ*_T1_ = 0.65, *γ*_T2_ = 0.68, *γ*_T3_ = 0.69), with moderate associations between depersonalization and lower personal accomplishment (*γ*_T1_=-0.22, *γ*_T2_=-0.14, *γ*_T3_=-0.24).

**Figure 4:**
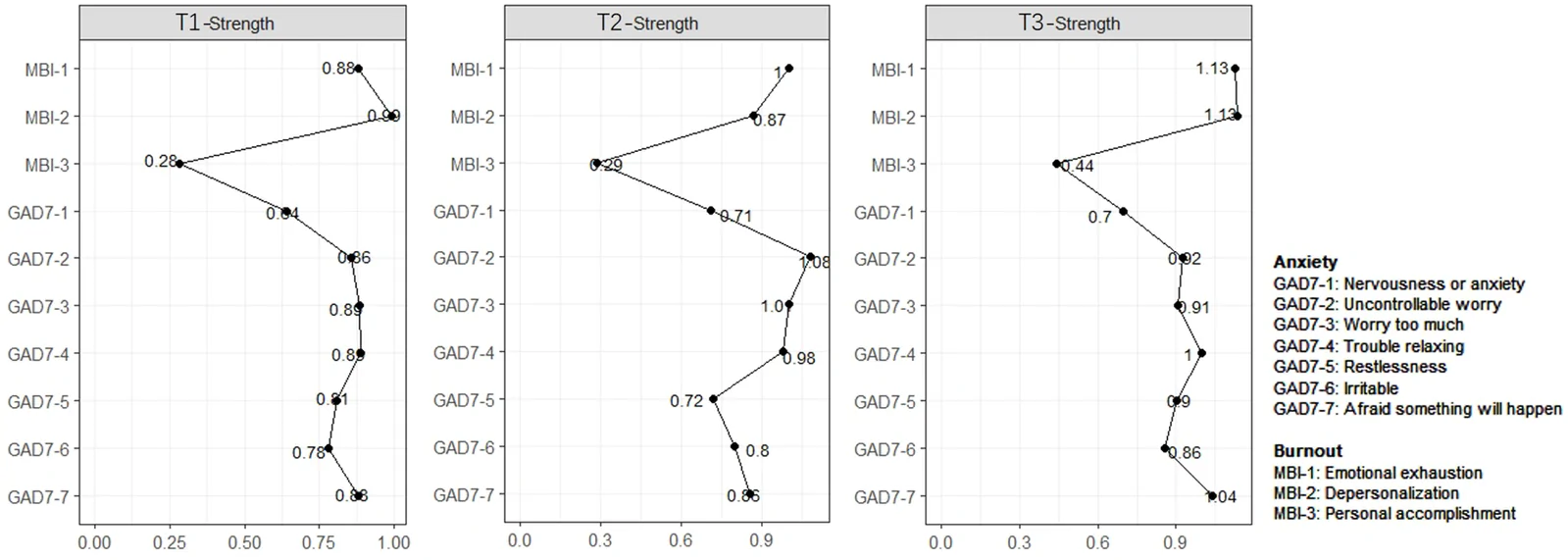
Centrality indices of contemporaneous networks for anxiety symptoms and burnout at T1, T2, and T3. Anxiety: GAD7-1: Nervousness or anxiety; GAD7-2: Uncontrollable worry; GAD7-3: Worry too much; GAD7-4: Trouble relaxing; GAD7-5: Restlessness; GAD7-6: Irritable; GAD7-7: Afraid something will happen; Burnout: MBI-1: Emotional exhaustion; MBI-2: Depersonalization; MBI-3: Personal accomplishment.

For anxiety centrality (strength ≥1), no core symptoms emerged at T1. At T2, “ uncontrollable worry” and “worry too much” were central, while at T3, “ afraid something will happen “ and “trouble relaxing” became central (**Figure 4**). For burnout, depersonalization was central at T1, emotional exhaustion at T2, and both dimensions were central at T3.

**Figure 5:**
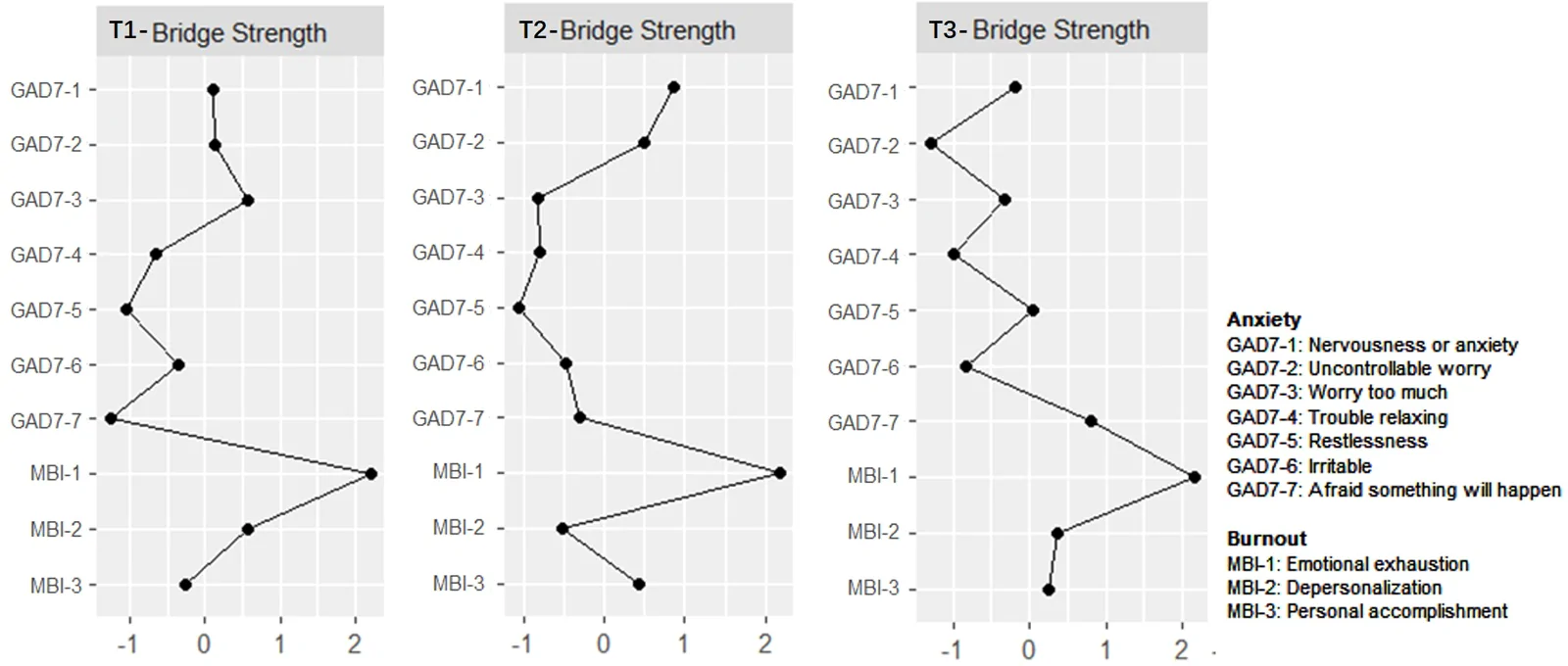
Centrality indices of bridging symptoms in contemporaneous networks at T1, T2, and T3. Anxiety: GAD7-1: Nervousness or anxiety; GAD7-2: Uncontrollable worry; GAD7-3: Worry too much; GAD7-4: Trouble relaxing; GAD7-5: Restlessness; GAD7-6: Irritable; GAD7-7: Afraid something will happen; Burnout: MBI-1: Emotional exhaustion; MBI-2: Depersonalization; MBI-3: Personal accomplishment.

“Emotional exhaustion” showed relatively high bridge strength in the descriptive centrality results. At T1, it was connected to “worry too much”, and at T2 and T3, it linked with “nervousness or anxiety” and “irritable” (**Figure 5**). “Uncontrollable worry” and “personal accomplishment” were linked at T1 and T2. At T3, “restlessness” and “afraid something will happen” were negatively associated with personal accomplishment.

Network stability analyses showed good strength stability (*CS*_T1_ = 0.594; *CS*_T2_ = 0.751; *CS*_T3_ = 0.594). Bridge strength stability was acceptable for T1–T2 (*CS*_T1&T2_ = 0.516) but poor for T3 (*CS*_T3_ = 0.128). Network structure comparisons showed no significant differences between T1–T2 (*M* = 0.18, *p* = 0.65), T2–T3 (*M* = 0.17, *p* = 0.57), or T1–T3 (*M* = 0.16, *p* = 0.78).

### 3.3 Cross-lagged networks of anxiety and burnout across time

**Figure 6:**
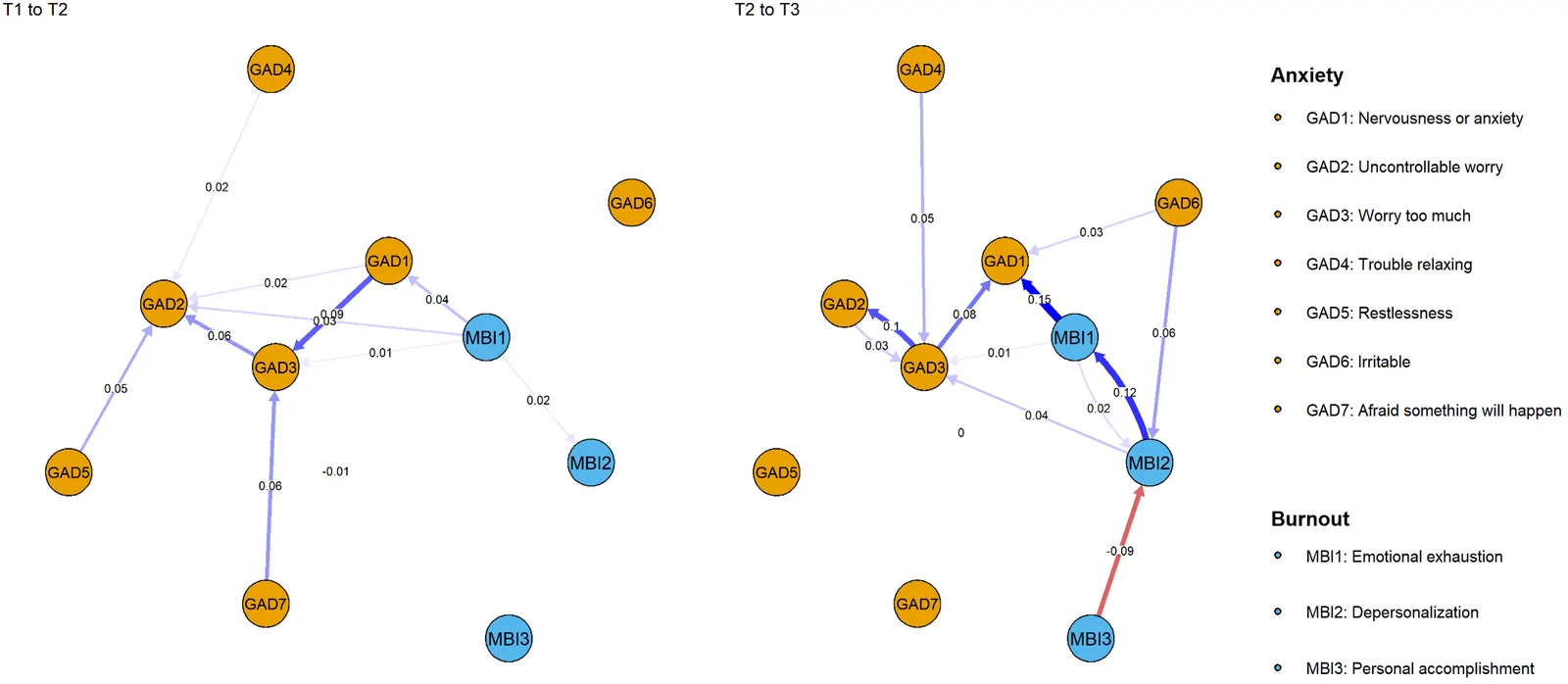
Cross-lagged networks of anxiety symptoms and burnout from T1→T2 (July 2023→January 2024) and T2→T3 (January 2024→July 2024). Networks with autoregressive edges are shown in **Supplementary Figure 1**.

**Figure 6** shows cross-lagged networks from T1 to T2 and T2 to T3. In the T1-to-T2 network, the cross-lagged associations were generally weak. Within anxiety symptoms, several earlier anxiety symptoms were linked to subsequent worry-related symptoms. Specifically, “restlessness” at T1 had a positive association with “uncontrollable worry” at T2 (*β* = 0.05), and “afraid something will happen” at T1 positively related to “worry too much” at T2 (*β* = 0.06). “Nervousness or anxiety” at T1 also showed a positive association with subsequent “worry too much” at T2 (*β* = 0.09). Among burnout dimensions, emotional exhaustion at T1 positively related to “nervousness or anxiety” at T2 (*β* = 0.04) and showed a weak positive association with depersonalization at T2 (*β* = 0.02).

For T2-to-T3 network, more cross-lagged associations between anxiety symptoms and burnout dimensions were observed. The strongest cross-dimensional predictive pathway emerged from “emotional exhaustion” at T2 to “nervousness or anxiety” at T3 (*β* = 0.15). Meanwhile, irritability at T2 also positively predicted depersonalization at T3 (*β* = 0.06). Within burnout dimensions, “depersonalization” at T2 positively predicted “emotional exhaustion” at T3 (*β* = 0.12), whereas personal accomplishment at T2 negatively predicted depersonalization at T3 (*β* = -0.09), suggesting that higher personal accomplishment was associated with lower subsequent depersonalization. Within anxiety symptoms, “uncontrollable worry” at T2 positively predicted “worry too much” at T3 (*β* = 0.10), and “worry too much” at T2 positively predicted “nervousness or anxiety” at T3 (*β* = 0.08).

In addition, the three burnout dimensions showed moderate autoregressive stability across waves: personal accomplishment (*β*_T1-T2_ = 0.28, *β*_T2-T3_ = 0.33), emotional exhaustion (*β*_T1-T2_ = 0.23, *β*_T2-T3_ = 0.37), and depersonalization (*β*_T1-T2_ = 0.18, *β*_T2-T3_ = 0.30). **Supplementary Figure 1** illustrated the network analysis model incorporating all autoregressive edges.

**Figure 7:**
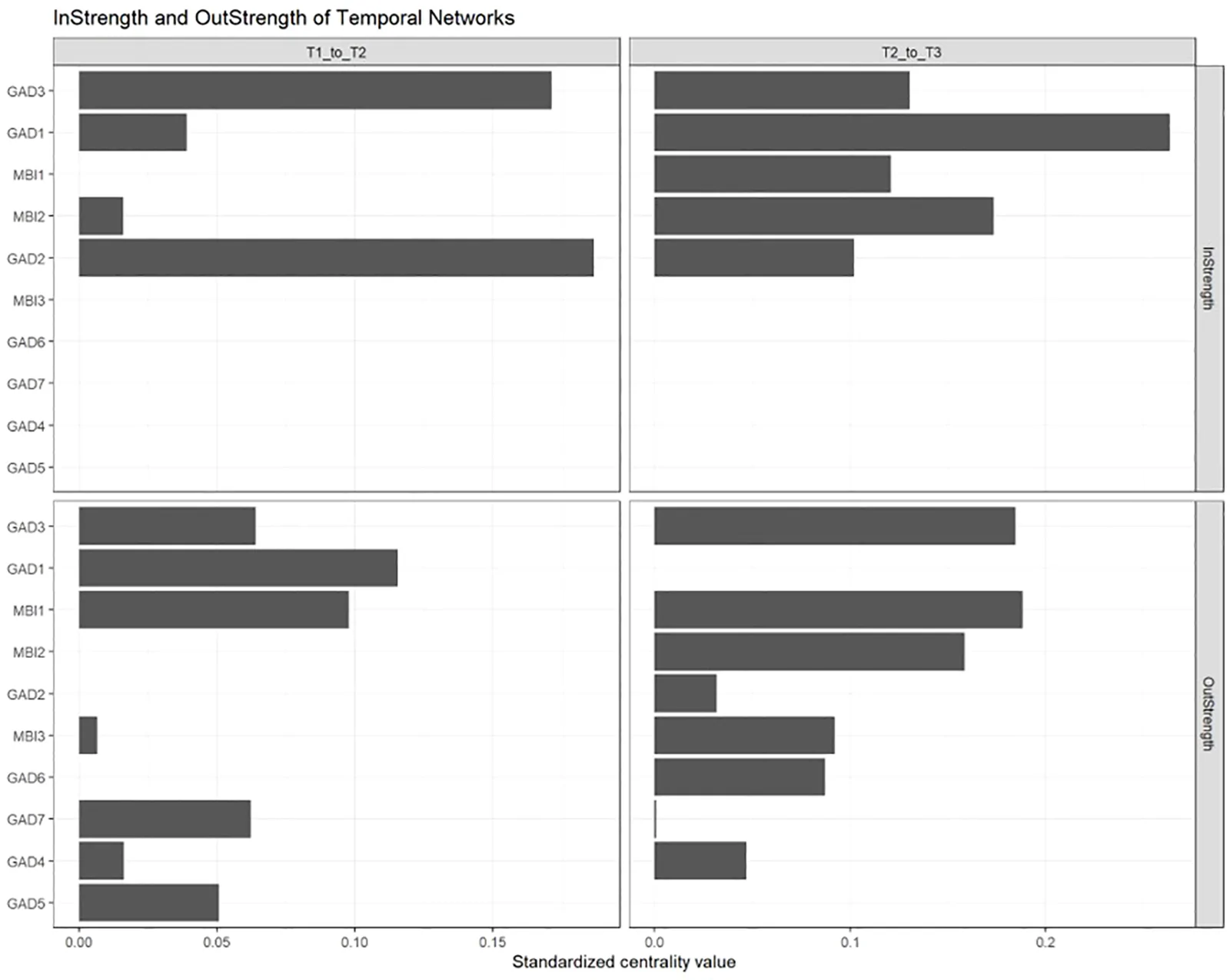
Centrality indices (outStrength and inStrength) of temporal networks from T1→T2 and T2→T3. Anxiety: GAD1: Nervousness or anxiety; GAD2: Uncontrollable worry; GAD3: Worry too much; GAD4: Trouble relaxing; GAD5: Restlessness; GAD6: Irritable; GAD7: Afraid something will happen; Burnout: MBI1: Emotional exhaustion; MBI2: Depersonalization; MBI3: Personal accomplishment.

Centrality analyses (**Figure 7**) further identified nodes with relatively high in-strength and out-strength in the temporal networks. In the T1-to-T2 network, GAD2 (uncontrollable worry) and GAD3 (worry too much) showed the highest in-strength values, indicating that these two worry-related symptoms were more likely to be predicted by other symptoms at the previous time point. For out-strength, GAD1 (nervousness or anxiety), MBI1 (emotional exhaustion), GAD3 (worry too much), and GAD7 (afraid something will happen) showed relatively higher values, suggesting that these symptoms or dimensions had greater predictive influence on other subsequent nodes. In the T2-to-T3 network, GAD1 (nervousness or anxiety) showed the highest in-strength, followed by MBI2 (depersonalization), GAD3 (worry too much), and MBI1 (emotional exhaustion). Regarding out-strength, MBI1 (emotional exhaustion), GAD3 (worry too much), and MBI2 (depersonalization) showed relatively higher values. Overall, the T2-to-T3 network showed a more distributed pattern of predictive influence than the T1-to-T2 network, with both anxiety symptoms and burnout dimensions contributing to later network activation.

**Figure 8:**
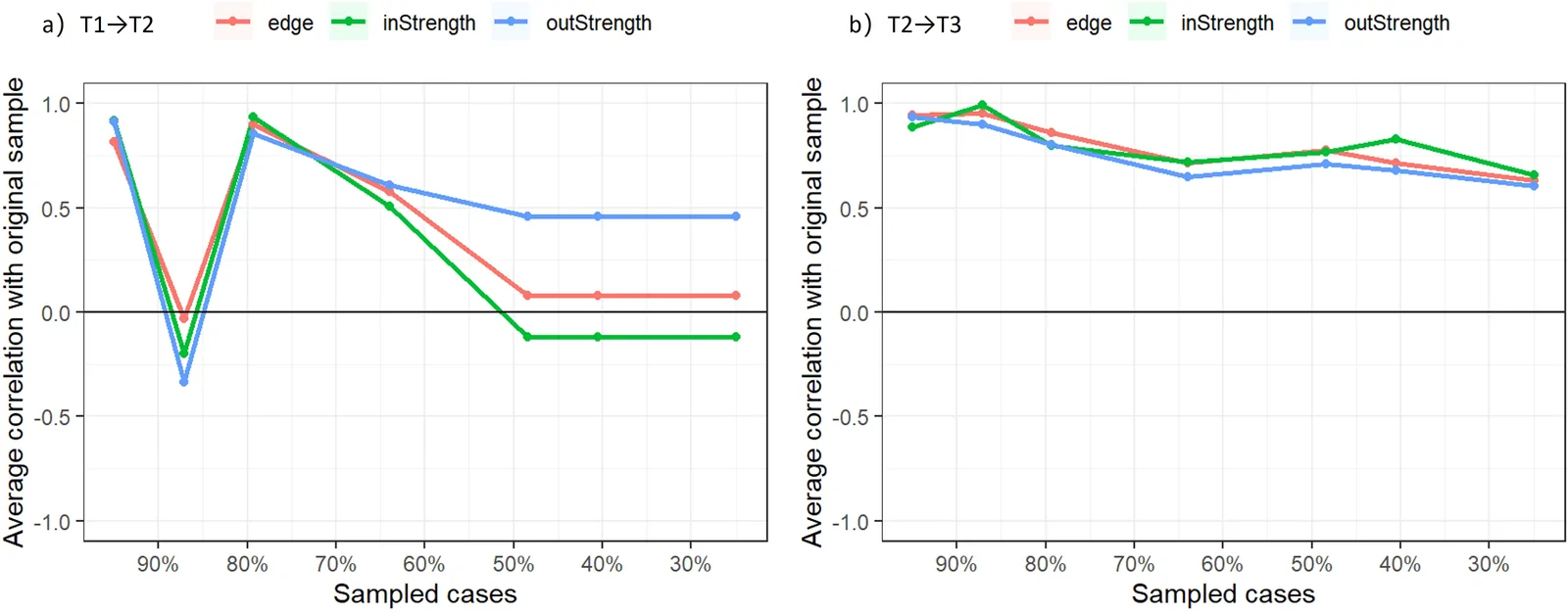
Stability Analysis of temporal network for **(a)** T1→T2 and **(b)** T2→T3.

Case-dropping bootstrap stability analyses (**Figure 8**) revealed weak overall network robustness for the T1→T2 model. For T2→T3, stability was marginally acceptable, with in-prediction *CS* = 0.594 (95% *CI* = [0.516, 0.751]) and out-prediction *CS* = 0.516 (95% *CI* = [0.360, 0.594]). Sensitivity analyses showed that the temporal networks were generally stable after removing demographic covariates. Using lambda.min produced denser networks. The number of nonzero edges increased from 11 to 47 (covariate-adjusted) and 54 (no covariates) in T1-to-T2, and from 13 to 54 and 59 in T2-to-T3. However, all main-model edges were retained with consistent signs (**Supplementary Table 2**). Overall, core temporal associations were robust, though network density was sensitive to penalty selection.

## 4 Discussion

This study used longitudinal data to analyze contemporaneous and temporal networks of anxiety and burnout in newly hired nurses, identifying central and bridging symptoms, autoregressive effects, and time-varying characteristics.

### 4.1 Prevalence of anxiety and burnout in newly hired nurses

Anxiety prevalence (4.3%–14.4%) was lower than the national average for nurses (23.2%), while burnout detection rates (47.4%–54.7%) were consistent with previous studies in China (50.0%–64.5%) (2, 3). The high rates of burnout may be attributed to several reasons. First, from a professional standpoint, nurses, as frontline workers in the healthcare system, face heavy workloads and high pressure, making them prone to physical and mental exhaustion. Second, nurses have frequent and close contact with patients, which requires responding to various demands. However, newly hired nurses may struggle to manage these relationships effectively and rely on rigid, formulaic communication—patterns that correlate with depersonalization, a key aspect of burnout. Third, the societal recognition of the nursing profession is relatively insufficient, with nurses often occupying a lower social status than doctors. This disparity can reduce nurses’ sense of personal accomplishment at work.

### 4.2 Central symptoms and bridge symptoms in cross-sectional networks

The core symptoms of anxiety evolved over time: no clear core at T1, “uncontrollable worry” and “worry too much” at T2, and “afraid something will happen” and “trouble relaxing” at T3. This may be attributable to the initial period of employment, where unfamiliar work content and environment often make new hires feel uneasy and uncertain, resulting in a persistent state of worry. If these feelings persist and are not addressed, they may develop into more pronounced anxiety symptoms like “uncontrollable worry” and “worry too much”. As employment tenure lengthens and job proficiency improves, this “worried” mentality may gradually diminish. However, because of the high workload and rapid pace of clinical nursing, newly hired nurses may still experience difficulties adapting to their new roles and responsibilities. In this context, anxiety symptoms such as “afraid something will happen” and “trouble relaxing” may become more prominent after one year of employment. This is consistent with previous studies showing that nursing students tend to exhibit anxiety centered on “worry” (16), while practicing medical staff experience anxiety related to “trouble relaxing” and “restlessness” (13, 14). For burnout, emotional exhaustion and depersonalization were consistently central, aligning with findings from a Chinese nurse study on nurses (17).

In terms of bridge symptoms, “emotional exhaustion” emerged as the most critical bridge symptom across all time points. Initially, “emotional exhaustion” was primarily associated with the anxiety symptom of “worry too much”. At the 6-month and 12-month follow-ups, “nervousness or anxiety” and “ irritable” emerged as key bridging symptoms. Early in employment, exhaustion may be more closely related to anxiety and worry. Over time, as nurses continue to face high intensity, tension, irritability, and difficulties in adaptation, they may experience reduced work enthusiasm and greater energy depletion.

The centrality of “emotional exhaustion” as a bridge symptom aligns with findings from other studies, such as one conducted among Chinese psychiatrists, which suggested that addressing emotional exhaustion may help prevent burnout from evolving into more severe mental health issues (31). However, the centrality indices of bridge symptoms in this study were not highly robust, indicating the need for caution when generalizing these findings to other populations.

### 4.3 Time-varying characteristics of networks in cross-lagged panel models

To explore the time-lag effects and directed associations between anxiety symptoms and burnout dimensions, cross-lagged panel networks analyses were conducted. According to the temporal networks, the relationship between anxiety and burnout changed across the first year of employment, differing from prior research that a history of anxiety is a significant risk factor for high burnout (32). In the T1-to-T2 temporal network, the cross-lagged associations were mainly concentrated within anxiety symptoms. For example, early nervousness or anxiety, restlessness, and fear that something bad might happen were associated with later worry-related symptoms. This finding is consistent with previous evidence that newly employed nurses experience anxiety and work stress during the early transition period, with stress and anxiety peaking during the first months of employment (33). The transition shock theory also suggests that newly graduated nurses often experience anxiety, insecurity, and instability when entering professional practice for the first time (34). Therefore, during the initial stage of employment, newly hired nurses may still be adapting to unfamiliar clinical environments, work routines, role expectations, and interpersonal demands, and their emotional distress may first manifest as persistent worry and tension rather than immediately developing into occupational burnout.

In contrast, the T2-to-T3 temporal network showed more distinct temporal associations between anxiety symptoms and burnout dimensions. Findings suggested that accumulated exhaustion may further increase later anxiety, which aligned with cross- sectional network findings that emotional exhaustion acted as a central bridge symptom between burnout and anxiety. Meanwhile, irritability also showed a positive predictive association with subsequent depersonalization, suggesting potential reciprocal associations between anxiety and burnout. It also echoes previous network evidence among psychiatric nurses, which showed that burnout, anxiety, depression, and stress were closely interconnected in healthcare workers (35).

However, unlike previous cross-sectional evidence suggesting that anxiety may contribute to burnout through positive coping strategies and interpersonal relationship problems among Chinese nurses (36), our longitudinal findings suggested that burnout may also precede later anxiety symptoms during the later stage of employment. This discrepancy may be partly explained by differences in study design and participants. Wang et al. focused on practicing nurses with more work experience, whose anxiety may affect burnout through established interpersonal relationships and coping patterns (36). By contrast, our participants were newly hired nurses undergoing clinical transition. During this period, increasing clinical responsibility, high workload, and adaptation pressure may first contribute to emotional exhaustion, which may then deplete coping resources and heighten later tension and nervousness. Thus, the observed direction from burnout to anxiety in our study may reflect the cumulative burden of early clinical adaptation rather than a simple anxiety-to-burnout pathway.

Within burnout dimensions, depersonalization may precede later emotional depletion, and lower personal accomplishment may be associated with higher subsequent depersonalization. In addition, the three burnout dimensions showed strong autoregressive effects, indicating that burnout-related experiences tended to persist over time. This pattern is consistent with previous cross-lagged network evidence showing that emotional exhaustion, depersonalization, and personal accomplishment can predict their own recurrence (18). Taken together, these findings suggest that newly hired nurses who experience early emotional exhaustion, depersonalization, or reduced personal accomplishment may be at greater risk of persistent burnout difficulties, particularly when work-related stress accumulates and timely support is lacking. Therefore, helping newly hired nurses develop a sense of competence, achievement, and professional identity during their first year of employment may be important for preventing the progression of burnout.

In summary, our findings suggest that anxiety symptoms and burnout dimensions may become more closely connected as newly hired nurses progress through the first year of clinical adaptation. Therefore, stage-specific support may be needed. During the early stage of employment, interventions should primarily focus on reducing anxiety and uncertainty through mentorship, structured orientation training, stress management, and psychological counseling (37). As nurses gradually adapt to clinical work, greater attention should be paid to early signs of occupational burnout, such as emotional exhaustion, depersonalization, and reduced personal accomplishment. Timely burnout-focused interventions, including workload support, career planning, professional identity development, and strategies to enhance personal accomplishment, may help prevent the progression from emotional detachment to more severe burnout and anxiety symptoms.

### 4.4 Innovations and limitations

This study innovatively applied a cross-lagged network model to analyze the associations and predictive directions of anxiety and burnout among newly hired nurses, using longitudinal data from three time points. Nonetheless, some limitations should be acknowledged. First, all anxiety and burnout measures were self-reported, which may introduce recall bias and social desirability bias. Second, the study was conducted in a single tertiary hospital in Sichuan Province, and the local occupational context, hospital management system, and training arrangements for newly hired nurses may limit the generalizability of the findings. Third, important work-related variables, such as department assignment, shift work, workload, nurse–patient conflicts, perceived organizational support, mentorship quality, and coping strategies, were not measured. Therefore, the mechanisms underlying the observed temporal associations could not be fully examined (38–41).

## 5 Conclusion

This study provides valuable insights into the dynamic relationships between anxiety symptoms and burnout dimensions among newly hired nurses during their first year of clinical work. Using contemporaneous and cross-lagged panel network analyses, we found that anxiety and burnout showed time-varying network patterns. In the contemporaneous networks, worry-related anxiety symptoms, trouble relaxing, and fear that something bad might happen emerged as important anxiety symptoms at different time points, while emotional exhaustion and depersonalization were central burnout dimensions. Emotional exhaustion also served as a bridge between anxiety and burnout, suggesting its potential role in linking psychological distress with occupational burnout.

The temporal networks further showed that the relationship between anxiety and burnout changed across the first year of employment. Early associations were mainly within anxiety symptoms, indicating that distress initially manifests as worry and tension. In the later stage, anxiety and burnout became more interconnected, with emotional exhaustion predicting later anxiety and depersonalization predicting subsequent emotional exhaustion. Lower personal accomplishment was linked to higher later depersonalization, and burnout dimensions showed strong persistence over time. These findings highlight the need for stage-specific support: early interventions should reduce anxiety and uncertainty, while later efforts should focus on identifying and managing burnout, which may help newly hired nurses reduce anxiety and prevent burnout escalation.

## Data Availability

The raw data supporting the conclusions of this article will be made available by the authors, without undue reservation.
