# Supplementary material for: Longitudinal relationship between anxiety symptoms and occupational burnout dimensions among newly hired nurses: a cross-lagged network analysis
Source: Front Psychiatry. 2026 Aug 7;17:1884139. doi: 10.3389/fpsyt.2026.1884139 (PMC13495780; doi:10.3389/fpsyt.2026.1884139)
Supplement: Supplementary file 1 [file SupplementaryFile1.docx]

Supplementary Table 1. Descriptive statistics of anxiety and burnout dimensions across three time points (T1-T3).

| Variable | T1 | T2 | T3 |
| --- | --- | --- | --- |
| Anxiety score,  Median (IQR)* | 0.00 (0.00, 1.00) | 1.00 (0.00, 2.00) | 1.00 (0.00, 2.00) |
| Emotional exhaustion,  Median (IQR)* | 10.00 (6.00, 16.00) | 12.00 (7.00, 18.00) | 12.00 (6.00, 18.00) |
| Depersonalization,  Median (IQR)* | 4.00 (1.00, 7.00) | 4.00 (2.00, 8.00) | 4.00 (2.00, 8.00) |
| Personal accomplishment,  Mean $\pm$SD^#^ | 26.57 ± 8.44 | 26.37 ± 9.12 | 28.07 ± 8.96 |

* Anxiety score (GAD-7 total score), emotional exhaustion, and depersonalization exhibited significant skewed distributions, hence presented as median (interquartile range, IQR).

**^#^** The personal accomplishment dimension approximated normal distribution and was summarized as mean ± standard deviation (SD).

| Supplementary Table 2. Correlation matrix of study variables (N = 397). | | | | | | | | | | | | | |
| --- | --- | --- | --- | --- | --- | --- | --- | --- | --- | --- | --- | --- | --- |
| Variables | M$\pm$SD | 1 | 2 | 3 | 4 | 5 | 6 | 7 | 8 | 9 | 10 | 11 | 12 |
| 1. T1-Anxiety | 0.90$\pm1.53$ | - |  |  |  |  |  |  |  |  |  |  |  |
| 2. T1-Burnout-1 | 11.53$\pm7.27$ | 0.36^**^ | - |  |  |  |  |  |  |  |  |  |  |
| 3. T1- Burnout -2 | 4.59$\pm3.68$ | 0.34^**^ | 0.75^**^ | - |  |  |  |  |  |  |  |  |  |
| 4. T1- Burnout -3 | 26.57$\pm8.44$ | -0.12^*^ | -0.19^**^ | -0.31^**^ | - |  |  |  |  |  |  |  |  |
| 5. T2-Anxiety | 1.67$\pm2.40$ | 0.38^**^ | 0.27^**^ | 0.26^**^ | -0.16^**^ | - |  |  |  |  |  |  |  |
| 6. T2- Burnout -1 | 12.89$\pm8.32$ | 0.26^**^ | 0.48^**^ | 0.40^**^ | -0.15^**^ | 0.48^**^ | - |  |  |  |  |  |  |
| 7. T2- Burnout -2 | 5.09$\pm4.12$ | 0.22^**^ | 0.42^**^ | 0.46^**^ | -0.17^**^ | 0.39^**^ | 0.77^**^ | - |  |  |  |  |  |
| 8. T2- Burnout -3 | 26.37$\pm9.12$ | -0.12^*^ | -0.19^**^ | -0.26^**^ | 0.47^**^ | -0.22^**^ | -0.14^**^ | -0.22^**^ | - |  |  |  |  |
| 9. T3-Anxiety | 1.58$\pm2.13$ | 0.46^**^ | 0.31^**^ | 0.31^**^ | -0.14^**^ | 0.45^**^ | 0.36^**^ | 0.37^**^ | -0.20^**^ | - |  |  |  |
| 10. T3- Burnout -1 | 12.91$\pm8.25$ | 0.29^**^ | 0.53^**^ | 0.42^**^ | -0.21^**^ | 0.35^**^ | 0.62^**^ | 0.56^**^ | -0.21^**^ | 0.41^**^ | - |  |  |
| 11. T3- Burnout -2 | 4.89$\pm3.94$ | 0.25^**^ | 0.48^**^ | 0.51^**^ | -0.29^**^ | 0.35^**^ | 0.44^**^ | 0.51^**^ | -0.32^**^ | 0.44^**^ | 0.77^**^ | - |  |
| 12. T3- Burnout -3 | 28.07$\pm8.96$ | -0.15^**^ | -0.16^**^ | -0.26^**^ | 0.45^**^ | -0.16^**^ | -0.18^**^ | -0.23^**^ | 0.55^**^ | -0.31^**^ | -0.25^**^ | -0.38^**^ | - |

** *p* < 0.01; * *p* < 0.05. Burnout-1= emotional exhaustion; Burnout -2= depersonalization; Burnout -3= personal accomplishment(reverse-scored). These are not partial correlations.

Supplementary Table 3. Sensitivity analyses for cross-lagged panel network models

| Wave | Sensitivity model | Pearson edge correlation | Spearman edge correlation | Main nonzero edges | Sens nonzero edges | Common nonzero edges | Jaccard index | Sign consistency common edges |
| --- | --- | --- | --- | --- | --- | --- | --- | --- |
| T1→T2 | Covariates + lambda.min | 0.62 | 0.56 | 11 | 47 | 11 | 0.23 | 1 |
| T1→T2 | No cov. + lambda.1se | 1.00 | 0.96 | 11 | 12 | 11 | 0.92 | 1 |
| T1→T2 | No cov. + lambda.min | 0.61 | 0.54 | 11 | 54 | 11 | 0.20 | 1 |
| T2→T3 | Covariates + lambda.min | 0.67 | 0.52 | 13 | 54 | 13 | 0.24 | 1 |
| T2→T3 | No cov. + lambda.1se | 1.00 | 1.00 | 13 | 13 | 13 | 1.00 | 1 |
| T2→T3 | No cov. + lambda.min | 0.64 | 0.51 | 13 | 59 | 13 | 0.22 | 1 |

Supplementary Figure 1. Cross-lagged networks of anxiety symptoms and burnout incorporating autoregressive edges

Anxiety: GAD1 (Nervousness or anxiety), GAD2 (Uncontrollable worry), GAD3 (Worry too much), GAD4 (Trouble relaxing), GAD5 (Restlessness), GAD6 (Irritable), GAD7 (Afraid something will happen);

Burnout: MBI1 (Emotional exhaustion), MBI2 (Depersonalization), MBI3 (Personal accomplishment)
